# Minilaparotomy-assisted approach for fetoscopic myelomeningocele closure

**DOI:** 10.3171/2026.1.FOCVID25234

**Published:** 2026-04-01

**Authors:** Katherine Holste, Scott Shainker, Farokh Demehri, Eyal Krispin, Alireza Shamshirsaz, Weston Northam

**Affiliations:** 1 Department of Neurosurgery,; 2 Division of Fetal Medicine and Surgery, and; 3 Department of Surgery, Boston Children’s Hospital, Boston, Massachusetts

**Keywords:** fetoscopic, myelomeningocele, fetal

## Abstract

Fetal closure has demonstrated a reduction in need for ventricular shunting, reversal of hindbrain herniation, and improved mobility compared to postnatal closure. Open fetal closure can benefit the fetus, but also places the mother at risk of obstetrical complications. Fetoscopic repair allows for prenatal closure of the myelomeningocele defect, while avoiding the open hysterotomy and providing the option for vaginal delivery in some cases. There are multiple techniques for performing fetoscopic repair; each has its benefits and detriments. Here a technique is presented for a minilaparotomy-assisted approach for fetoscopic myelomeningocele closure.

The video can be found here: https://stream.cadmore.media/r10.3171/2026.1.FOCVID25234

## Transcript

### 0:20 Introduction.

Here we present a minilaparotomy-assisted approach for fetoscopic myelomeningocele closure. A 22-year-old G1P0 woman presented for evaluation of an open neural tube defect. She was evaluated in our multidisciplinary clinic by maternal fetal medicine, pediatric surgery, pediatric neurosurgery, and neonatology. Fetal MRI demonstrated a large myelomeningocele defect starting at L4, which was confirmed with ultrasound. The fetus also had a Chiari II malformation with hindbrain herniation extending down to approximately C2 level. There was no ventriculomegaly present preoperatively. The preoperative ventricular size was 6.7 mm unilaterally when measured at the level of the ventricular atrium. Genetic testing and amniocentesis were performed, which showed no obvious anomalies. Excellent lower extremity movement was observed including plantarflexion. The placenta was located posteriorly. Our institution follows the general guidelines of the MOMS trial criteria for patient selection for fetal closure and, therefore, she was felt to be a good candidate for fetal surgery. Because the placenta was located posteriorly, and she was planning on having subsequent children including the current pregnancy via vaginal delivery and wanted to avoid a larger laparotomy, she was felt to be a good candidate for fetal closure using a minilaparotomy approach.

### 1:36 Fetal Anesthesia.

Fetal anesthesia is delivered after induction of maternal anesthesia using an intramuscular combination of rocuronium, fentanyl, and atropine. Fetal heart rate was monitored every 5 minutes by the primary surgical team. After administration of fetal anesthetic, amnioinfusion of warmed lactated Ringer’s was used to create space within the uterus for safe trochar placement and was done prior to port placement.

### 2:01 Minilaparotomy.

A 4-cm infraumbilical midline vertical incision was made for initial port placement. Four circumferential "box sutures" are placed around the 5-mm port site before it is inserted using 3-0 PDS suture to incorporate the membranes. An ultrasound is then used to place a 5-mm port using Seldinger technique for the endoscope. Next, two 4-mm ports were inserted percutaneously and the uterus was insufflated with CO_2_. Insufflation pressures ranged from 8–17 mm Hg.

### 2:38 Placode Dissection.

Using the laparoscopic graspers and scissors with monopolar cautery, a linear opening was made into the myelomeningocele sac at the rostral end of the defect, which was positioned closest to the scope. The subarachnoid space was entered with egress of CSF. A plane was developed lateral to the border of the placode, releasing it first on the left side and then on the right using the scissors with occasional monopolar cautery. The rostral and caudal ends of the placode were released, and the placode dropped into the spinal canal nicely. All dermal elements were removed from the placode and any arachnoid adhesions were released.

### 3:15 Suprafascial Dissection.

The scissors were then used to enter into the normal skin rostrally and caudally to identify the underlying epidural plane. Nonviable skin was trimmed off in the transition zone. Next, extensive suprafascial dissection was performed to relax the skin edges to help with closure. This was performed laterally until the flanks were reached as well as rostrally and caudally.

### 3:38 Expansile Duraplasty.

Next an expansive AlloDerm duraplasty was done based on the preoperative ultrasound measurements. AlloDerm is the most commonly used duraplasty material at our institution as it is versatile and strong enough to suture and be pulled down through the ports with laparoscopic graspers. Allograft duraplasty is more efficient than primary dural closure at our center when performing a fetoscopic closure and yields an expansion of the thecal sac. A double-armed 4-0 barbed suture was loaded into the graft at the rostral pole and another barbed suture at the caudal pole. The graft was loaded into the 5-mm port. The caudal pole was secured first and then the double-armed suture was run on either side of the graft toward the caudal end.

### 4:25 Skin Closure and Removal of Ports.

Next, the skin was closed with a running barbed suture, first through the middle of the incision and running each arm rostrally and caudally. The suture was then slowly cinched to approximate the skin edges. The port sites were closed using the ability to move the minilaparotomy over each of the port sites to ensure the membranes were reapproximated.

The uterine cavity was then irrigated with warm lactated Ringer’s solution, and an amniotic fluid exchange was performed to maintain a relatively constant uterine size. Ultrasound revealed no fetal membrane separation. The mother and fetus tolerated the procedure well. Fetal heart rate pre- and postprocedure was 125 and 136 bpm, respectively, with a single period of bradycardia noted to 110 and spontaneously improved over 5–10 minutes.

### 5:12 Clinical Outcome.

The baby was born at 34 weeks and 2 days due to premature rupture of membranes via c-section due to breech position and tolerated his stay in the NICU well. The skin was well healed on birth. MRI brain and spine performed showed minimal ventriculomegaly and good reversal of the Chiari II malformation. He has been followed for 1.5 years without needing treatment for hydrocephalus. Functionally, he is able to bear weight well when holding onto furniture but has required AFO braces. He has antigravity dorsiflexion and intermittent plantarflexion.

### 5:43 Discussion.

The minilaparotomy approach is useful in patients with a posterior placenta who are planning to have subsequent children including their current pregnancy via vaginal delivery and want to avoid a larger laparotomy. The minilaparotomy approach may be used in select cases to allow for the robust closure of percutaneously placed port sites during fetoscopic repair. This approach is contraindicated if the placenta is located anteriorly. Positioning of the fetus can be difficult as the fetus cannot be manually reoriented as it can be when the uterus is exteriorized. More care is needed when passing rigid instruments through a flexible trochar that traverses multiple subcutaneous anatomical planes. It is more difficult, and there is a learning curve when placing the ports. Higher BMI can require greater planning for appropriate port placement but is not a contraindication to this approach.

Fetoscopic repair of neural tube defects aims to provide the same benefit to the fetus as open repair, as shown by the MOMs trial, while minimizing maternal morbidities. Across studies of prenatal neural tube defect repair, the prevalence of chorioamniotic membrane separation is comparable between open and fetoscopic approaches. The gestational age at birth in this case is similar to previously published fully percutaneous techniques, mean age 33 weeks gestation, as well as open fetal repair at the mean age of 34 weeks gestation, but is younger than one other combined laparotomy-assisted two-port approach where the uterus is exteriorized. Minilaparotomy may be used in select cases to allow for the robust closure of percutaneously placed port sites during fetoscopic repair.

### 7:16 References.^1–7^

## Disclosures

The authors report no conflict of interest concerning the materials or methods used in this study or the findings specified in this publication.

## Author Contributions

Primary surgeon: Northam, Krispin, Shamshirsaz. Assistant surgeon: Shainker, Demehri. Editing and drafting the video and abstract: Northam, Holste, Demehri, Krispin. Critically revising the work: Northam, Holste, Shainker, Demehri, Krispin. Reviewed submitted version of the work: Northam, Holste, Shainker, Demehri, Krispin. Approved the final version of the work on behalf of all authors: Northam. Supervision: Krispin.

## Supplemental Information

### Patient Informed Consent

The necessary patient informed consent was obtained in this study.
